# Low Expression of CAPON in Glioma Contributes to Cell Proliferation via the Akt Signaling Pathway

**DOI:** 10.3390/ijms17111859

**Published:** 2016-11-18

**Authors:** Shangfeng Gao, Jie Wang, Tong Zhang, Guangping Liu, Lei Jin, Daofei Ji, Peng Wang, Qingming Meng, Yufu Zhu, Rutong Yu

**Affiliations:** 1 Institute of Nervous System Diseases, Xuzhou Medical University, 84 West Huai-Hai Road, Xuzhou 221002, Jiangsu, China; gaoshangfeng@xzhmu.edu.cn (S.G.); yunfan661@163.com (Q.M.); 2 Brain Hospital, Affiliated Hospital of Xuzhou Medical University, 99 West Huai-Hai Road, Xuzhou 221002, Jiangsu, China; jiew871@163.com (J.W.); zhtfxl@163.com (T.Z.); raycooper@126.com (L.J.); 15152118415@163.com (D.J.); Angelover1314520@163.com (P.W.); 3 People’s Hospital of Juxian, 100 Fulaizhong Road in Juxian, Rizhao 276500, Shandong, China; lgp123456002@163.com

**Keywords:** CAPON, glioma, proliferation, Akt, mTOR

## Abstract

CAPON is an adapter protein for nitric oxide synthase 1 (NOS1). CAPON has two isoforms in the human brain: CAPON-L (long form of CAPON) and CAPON-S (short form of CAPON). Recent studies have indicated the involvement of CAPON in tumorigenesis beyond its classical role in NOS1 activity regulation. In this study, we found that the protein levels of CAPON-S, but not than CAPON-L, were significantly decreased in glioma tissues. Therefore, we established lentivirus-mediated stable cell lines with CAPON-S overexpression or down-regulation, and investigated the role of CAPON-S in the proliferation of glioma cells by using CCK8, EdU, and flow cytometry assays. Overexpression of CAPON-S reduced the cell variability and the percentage of EdU-positive cells, and arrested the cells in the G1 phase in glioma cells. Silencing of CAPON by short-hairpin RNA showed the opposite effects. Furthermore, an intracellular signaling array revealed that overexpression of CAPON-S resulted in a remarkable reduction in the phosphorylation of Akt and S6 ribosomal protein in glioma cells, which was further confirmed by Western blot. These findings suggest that CAPON may function as a tumor suppressor in human brain glioma and that the inactivation of the Akt signaling pathway caused by CAPON-S overexpression may provide insight into the underlying mechanism of CAPON in glioma cell proliferation.

## 1. Introduction

Glioma is the most common and aggressive tumor in the central nervous system [1]. It is characterized by uncontrolled proliferation, anti-apoptotic effect, invasion, and migration [2]. Tumor cells cannot be completely removed by surgery, radiotherapy, and chemotherapy, thus leading to recurrence after operation [3], short survival time, high mortality, and poor prognosis [4]. It was reported that the median survival of glioblastoma patients was only 12–15 months [5]. Therefore, in order to improve the treatment of this currently incurable cancer, it is necessary to search for novel therapeutic targets to combat tumor proliferation.

Nitric oxide (NO), an important bioactive molecule, is involved in regulating many physiological and pathological processes, such as vasodilation [6], neurotransmission [7], tumorigenesis [8], and host defense reactions [9]. In the nervous system, NO is produced from arginine predominantly by nitric oxide synthase 1 [10]. CAPON, an adapter protein of nitric oxide synthase 1 (also known as NOS1AP), was first identified in rat brain tissue in 1998 [11]. CAPON is widely expressed in a variety of tissues including the brain, cardiac muscle [12], skeletal muscle [13], and pancreas [14].

In the human brain, CAPON has two isoforms: the long form of CAPON (CAPON-L) and the short form of CAPON (CAPON-S) [15]. CAPON plays a significant role in many pathological processes such as cerebral ischemia reperfusion, peripheral nerve injury, and excitotoxicity [16]. A number of recent studies have suggested the possible involvement of CAPON in tumorigenesis. CAPON links to scribble, which in turn binds to Yes-associated protein (YAP) to form a ternary complex of CAPON-scribble-YAP [17]. Scribble is a polarity protein functioning in cell polarity, differentiation, and migration [18]. YAP is an effector of the Hippo signaling pathway that plays an important role in regulating cell proliferation, differentiation, and organ growth [19]. Both scribble and YAP are involved in tumor cell proliferation and migration [20,21], indicating the similar role of their partner, CAPON. Overexpressing CAPON restricted aberrant cell growth, whereas silencing CAPON promoted proliferation in breast cancer cells [17,22]. It seems that CAPON may be a potential tumor suppressor, although the related evidence is not sufficient at present.

It was previously reported that CAPON was located in the cytoplasm of cultured rat primary astrocytes [23]. In this study, we firstly assessed the expression changes of CAPON (including both CAPON-L and CAPON-S) in human glioma tissues and nontumor brain tissues and then investigated the role of CAPON in the proliferation of glioma cells. In addition, we screened the CAPON-S-overexpressing glioma cells to reveal the changes in some important signaling pathways related to tumorigenesis.

## 2. Results

### 2.1. Expression Changes of CAPON in Human Glioma Tissues

**Figure 1:**
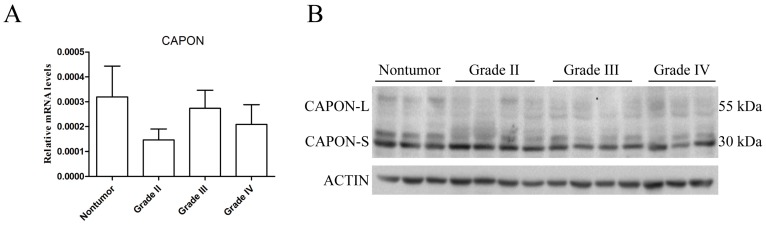

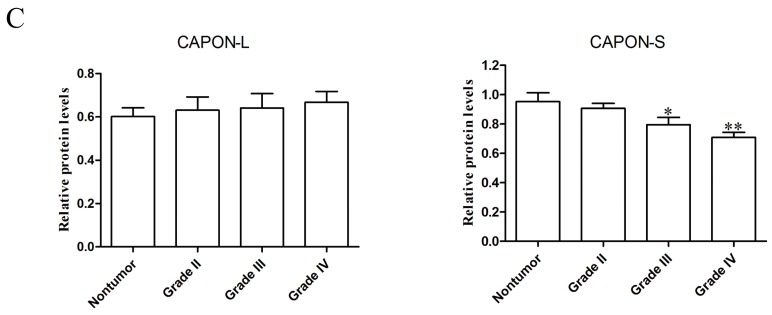
Changes in the expression levels of CAPON in nontumorous and glioma tissues. (**A**) Quantitative real-time PCR was used to measure the mRNA levels of CAPON in nontumor brain tissues (*n* = 8) and various grades of glioma tissues (*n* = 8 for each grade); (**B**,**C**) Western blot was used to measure the protein levels of CAPON in nontumor brain tissues (*n* = 9) and different grades of glioma tissues (Grade II, *n* =12; Grade III, *n* = 12; Grade IV, *n* = 9). Representative blot pictures are shown in **B** and quantitative graphs for relative CAPON-L and CAPON-S protein levels are shown in **C** (* *p* < 0.05; ** *p* < 0.01).

We used quantitative real-time PCR (qRT-PCR) to analyze the CAPON-mRNA 1evels in eight nontumorous brain tissues and 24 glioma specimens (*n* = 8 for each grade). There was no significant difference in the CAPON-mRNA levels between nontumor and glioma tissues with Grade II, III or IV (Figure 1A, all *p* > 0.05). We next employed Western blot to detect the CAPON protein levels in nine nontumorous brain tissues and 33 glioma specimens (*n* = 12 for Grade II, *n* = 12 for Grade III, and *n* = 9 for Grade IV). Since the CAPON antibody could recognize both CAPON-L (55 kDa) and CAPON-S (30 kDa) in the nontumorous human brain (Figure 1B), we quantitatively analyzed the expression levels of both isoforms. Interestingly, the protein levels of CAPON-S were significantly decreased in glioma Grade III (*p* = 0.049) and Grade IV (*p* = 0.002), while none of the glioma groups had significant changes in the CAPON-L protein levels (all *p* > 0.05) compared to the nontumor group (Figure 1C). These results indicated a low expression level of CAPON-S in glioma tissues.

### 2.2. CAPON-S Overexpression Efficiency and CAPON Down-Regulation Efficiency in Glioma Cells

**Figure 2:**
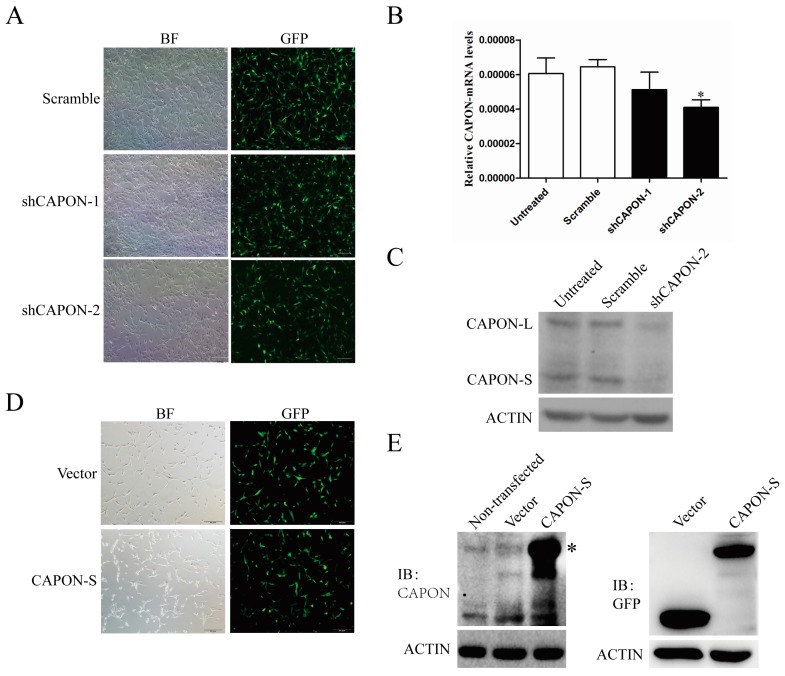
Identification of the efficiency of CAPON down-regulation and CAPON-S up-regulation in glioma cells. (**A**) The lentivirus infection efficiency was indicated by bright field (BF) and GFP fluorescence in scramble and shCAPON groups (Magnification ×100); (**B**) qRT-PCR analysis of the CAPON down-regulation efficiency at mRNA levels (* *p* < 0.05); (**C**) Western blot analysis of the down-regulation efficiency of shCAPON-2 at protein levels; (**D**) The lentivirus infection efficiency was indicated by bright field (BF) and GFP fluorescence in Vector group and CAPON-S group (Magnification ×100); (**E**) Western blot showed the overexpression efficiency of CAPON-S as detected by both CAPON and GFP antibodies. * indicate the overlap between CAPON-L and GFP-CAPON-S bands.

To evaluate the role of CAPON in glioma cell proliferation, we established lentivirus-mediated C6 cell lines with stable down-regulation of CAPON. Both CAPON shRNAs (short-hairpin RNA) showed an infection efficiency of more than 90% in C6 glioma cells, as indicated by GFP fluorescence (Figure 2A). qRT-PCR analysis revealed a reduction in the CAPON-mRNA levels by both shRNAs, and the shCAPON2 reached statistically differences (*p* = 0.038, Figure 2B). Western blot showed that both CAPON-L and CAPON-S protein levels were down-regulated by shCAPON-2 (Figure 2C), which was therefore used in the functional experiments. Moreover, we established lentivirus-mediated U87 cells lines with stable overexpression of CAPON-S. Fluorescence microscopy observation showed that 70% of lentivirus-infected U87 cells had GFP fluorescence (Figure 2D). Western blot performed with both CAPON and GFP antibodies further confirmed that the exogenous CAPON-S was abundantly overexpressed in U87 glioma cells (Figure 2E). It should be noted that the molecular weights of CAPON-L and GFP-CAPON-S are the same and their bands overlapped in the CAPON-S-overexpressing group. These data indicated that lentivius-mediated stable cell lines with CAPON down-regulation and CAPON-S overexpression were successfully established.

### 2.3. Effects of CAPON-S Overexpression or CAPON Down-Regulation on the Proliferation of Glioma Cells

**Figure 3:**
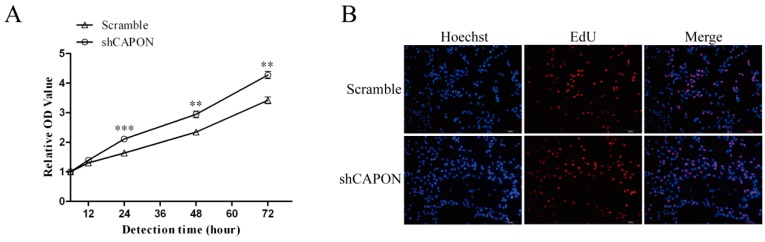

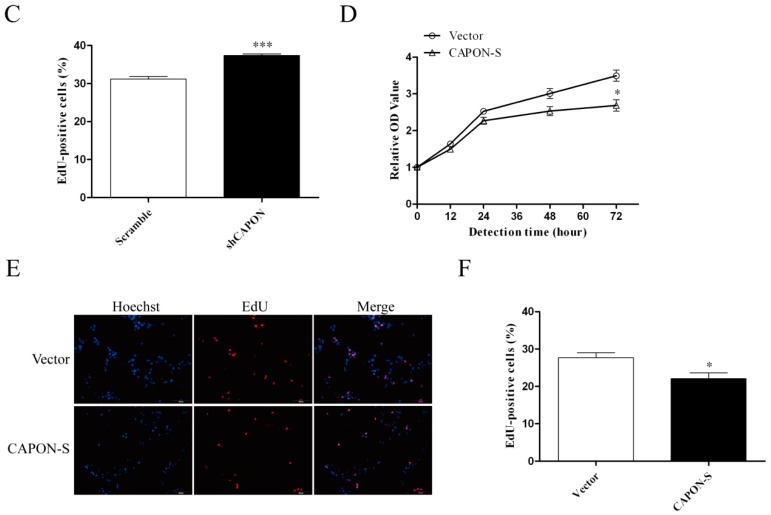
Influences of CAPON-S on the proliferation of glioma cells. (**A**) CCK8 assay was used to measure the cell viability in Scramble and shCAPON C6 glioma cells; (**B**,**C**) EdU assay was used to evaluate the proliferation of Scramble and shCAPON C6 glioma cells. Representative images for EdU-positive C6 glioma cells (red) and Hoechst-stained nuclei (blue) are shown in **B** (Magnification ×100). Quantification of the percentage of EdU-positive cells is shown in **C**; (**D**) CCK8 assay was used to assess the cell viability in Vector and CAPON-S U87 glioma cells; (**E**,**F**) EdU assay was used to evaluate the proliferation of Vector and CAPON-S U87 glioma cells. Representative images for EdU-positive U87 glioma cells (red) and Hoechst-stained nuclei (blue) are show in E (Magnification ×100). Quantification of the percentages of EdU-positive cells is shown in F. (* *p* < 0.05; ** *p* < 0.01; *** *p* < 0.001).

In glioma C6 cells, the CCK8 assay showed that silencing CAPON by shRNA significantly increased the cell viability at 24 h (*p* < 0.001), 48 h (*p* = 0.001) and 72 h (*p* = 0.001) (Figure 3A). During DNA replication, 5-ethynyl-2′-deoxyuridine (EdU) is readily incorporated into cellular DNA. EdU assay is a method for measuring cell proliferation in the well-preserved cellular and chromatin ultrastructures. There was a significant increase in the percentage of EdU-positive cells in the shCAPON group compared to the Scramble group (*p* = 0.001, Figure 3B,C). These results indicated that the down-regulation of CAPON promoted the proliferation of C6 glioma cells. Moreover, overexpression of CAPON-S exhibited an inhibiting effect on glioma cell proliferation. For example, overexpressing CAPON-S remarkably decreased the cell viability at 48 h (*p* = 0.057) and 72 h (*p* = 0.021) (Figure 3D), and significantly reduced the percentage of EdU-positive cells in U87 glioma cells (*p* = 0.016, Figure 3E,F). These findings suggested that CAPON had a significant inhibitory effect on the proliferation of glioma cells.

### 2.4. Effects of CAPON-S Overexpression or CAPON Down-Regulation on the Cell Cycle Progression of Glioma Cells

**Figure 4:**
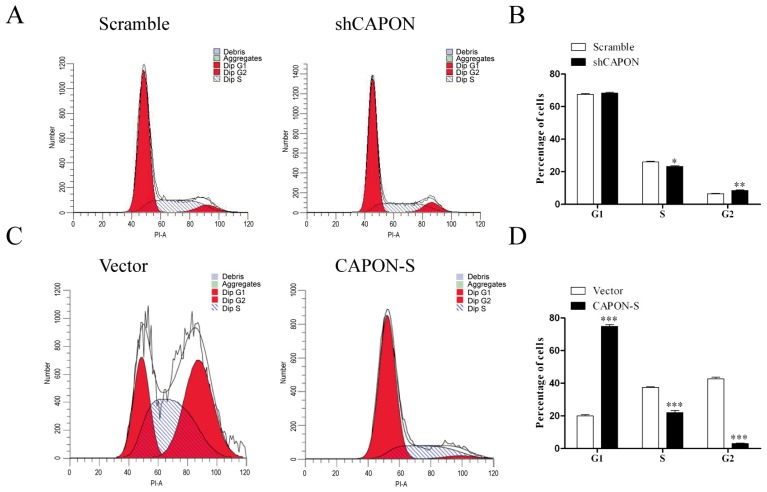
Effects of CAPON-S on the cell cycle progression in glioma cells. Flow cytometry was employed to analyze the cell cycle progression in CAPON-silencing C6 cells and CAPON-S-overexpressing U87 cells. Representative histograms for C6 and U87 cells are shown in **A** and **C**, respectively. The percentages of cells at each stage of cell cycle are shown in **B** (C6) and **D** (U87) (* *p* < 0.05; ** *p* < 0.01; *** *p* < 0.001).

Flow cytometry was used to assess the cell cycle progression in CAPON-silencing and CAPON-S-overexpressing glioma cells. Down-regulating CAPON resulted in more cells in the G2 phase (*p* = 0.009) and less cells in the S phase (*p* = 0.024) (Figure 4A,B). Overexpression of CAPON-S impeded the cell cycle progression in U87 cells. More cells were arrested in the G1 phase and the percentage of the cells in the S and G2 phases was significantly decreased (*p* < 0.001, Figure 4C,D). These results suggested that CAPON negatively regulated cell cycle progression in glioma cells.

### 2.5. Effects of CAPON-S Overexpression on the Important Intracellular Signaling Molecules in Glioma Cells

**Figure 5:**
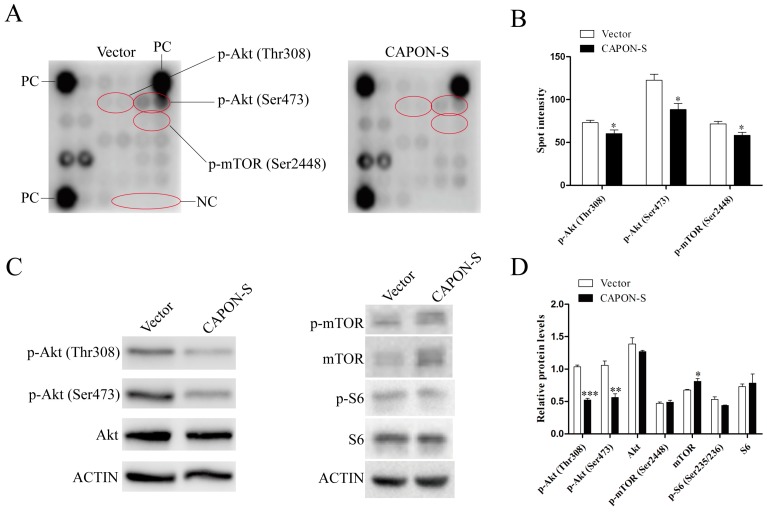
Regulation effect of overexpressing CAPON-S on the Akt and mTOR signaling. (**A**,**B**) Antibody array was used to screen important signaling molecules in Vector and CAPON-S U87 glioma cells. Representative spot pictures are shown in (**A**); PC, positive control; NC, negative control. The spot intensities of those molecules with significant changes, such as p-Akt (Thr308), p-Akt (Ser473), and p-mTOR (Ser2448), are shown in (**B**); (**C**,**D**) Western blot was used to confirm the antibody array data in Vector and CAPON-S U87 glioma cells; Representative blot pictures are shown in (**C**); Quantitative graphs for the relative protein levels normalized to Actin are shown in (**D**). (* *p* < 0.05; ** *p* < 0.01; *** *p* < 0.001).

In order to explore the candidate mechanisms in U87 glioma cells by CAPON-S stable overexpression, we performed an intracellular signaling array involving 18 important signaling molecules with phosphorylation or cleavage (Table S1). This array revealed that the phosphorylated Akt (*p* = 0.040 for Thr308; *p* = 0.015 for Ser473), S6 ribosomal protein (Ser235/236, *p* = 0.070) and mTOR (Ser2448, *p* = 0.024) were remarkably reduced in CAPON-S-overexpressing cells (Figure 5A,B). We then used Western blot to confirm the results. As shown in Figure 5C,D, overexpressing CAPON-S markedly deceased the level of p-Akt (Thr308, *p* < 0.001), p-Akt (Ser473, *p* = 0.005), and p-S6 (Ser235/236, *p* = 0.090), without affecting the total Akt and S6 level (all *p* > 0.05). However, the total mTOR level (*p* = 0.035), instead of the p-mTOR (Ser2448, *p* = 0.063) level, showed significant differences between the Vector and CAPON-S groups (Figure 5C,D). These data indicated that overexpression of CAPON-S led to the inactivation of the Akt signaling pathway.

## 3. Discussion

In this study, the expression of CAPON-S, the short form of CAPON, was significantly decreased in glioma tissues. Further functional experiments of stable overexpression or down-regulation demonstrated that CAPON had an inhibitory effect on the proliferation of glioma cells. In addition, we screened some important signaling molecules in CAPON-S-overexpressing cells and found that the Akt-S6 signaling was in the downstream of CAPON. These findings indicate that CAPON may serve as a tumor suppressor in glioma, possibly through inactivating the Akt signaling pathway.

CAPON, an adaptor protein for NOS1, is capable of disrupting the association of NOS1 with the scaffolding proteins PSD-93 and PSD-95 and may lead to decreased NO release [11]. NO regulates angiogenesis and the expression of cancer-related genes and may lead to the growth and metastasis of tumors [8]. The CAPON levels were reduced in the primary transfer type of breast cancer [22] and the low CAPON-S expression level was found in glioma tissues in our study. It is speculated that low CAPON levels may result in the higher NO production in cancer, which in turn promotes the tumor metastasis and growth. Due to the lack of survival data of our glioma patients, we performed Kaplan-Meier analysis in the R2 genomics database (available online: http://hgserver1.amc.nl/cgi-bin/r2/main.cgi). As expected, the low expression level of CAPON was associated with the poor survival of glioblastoma patients (Figure S1, *p* = 0.055). These findings suggested that low CAPON levels predicted a worse prognosis and that CAPON might serve as a promising biomarker in glioma.

CCK8 and EdU assays showed that silencing CAPON promoted the proliferation of glioma cells. The result was inconsistent with the previous studies in breast cancer cells. Down-regulation of CAPON enhanced anchorage-independent cell growth, whereas overexpression of CAPON inhibited basal and stimulated cell growth [17,22]. CAPON is not only bound to NOS1, but also linked to YAP though binding to scribble [17]. Silencing CAPON reduced the phosphorylation of YAP and the upstream kinase Lats1, while overexpression of CAPON led to the increase in the phosphorylated levels of YAP and Lats1 and the decreased YAP location to the nucleus [17]. These data implicated the role of CAPON in the regulation of core Hippo signaling and supported the idea that CAPON functioned as a tumor suppressor.

We also explored the candidate mechanism that CAPON inhibited the proliferation of glioma cells. Overexpression of CAPON-S significantly arrested the cells in the G1 phase. Thus, CAPON might inhibit glioma cell proliferation through cell cycle regulation. Moreover, an intracellular signaling array was applied to screen important signaling cascades in CAPON-S-overexpressing glioma cells. In comparison to p38MAPK inactivation in hippocampal neurons [24,25], overexpression of CAPON-S remarkably suppressed the phosphorylation of Akt (Thr308 and Ser473) and S6 (Ser235/236), in glioma cells, without affecting any signaling molecule in the MAPK pathway (Table S1). Interestingly, the decreased phosphorylation of mTOR (Ser2448) as found by antibody array was not confirmed by Western blot. Instead, overexpression of CAPON-S significantly increased the total mTOR level, suggesting the regulation of mTOR at the expression level, which deserves a closer look in the future. For the first time, we demonstrated the inhibitory effects of CAPON overexpression on the Akt signaling pathway, which is frequently activated in gliomagenesis [1].

A few limitations should be mentioned here. Firstly, CCK8 and EdU assays indicated that silencing CAPON showed more significant influences on glioma cell proliferation than the overexpression of CAPON-S (*p* < 0.01 vs. *p* < 0.05), verifying the contribution of CAPON-L to cell proliferation. However, we did not check the effects of overexpressing CAPON-L on the proliferation ability because the survival of glioma cells with stable CAPON-L overexpression was strongly affected. Secondly, we down-regulated CAPON expression in rat C6 cells and up-regulated CAPON-S in human U87 cells. Although a consistent conclusion was obtained in the cell lines from different species, further investigations are needed in other human glioma cell lines. In addition, it is necessary to perform brain xenograft experiments to confirm the inhibitory role of CAPON on glioma growth in vivo.

In conclusion, the protein expression level of CAPON-S in glioma tissues was lower than that in nontumorous brain tissues. Overexpression of CAPON-S inhibited the proliferation of the glioma cells, whereas down-regulation of CAPON promoted the proliferation of glioma cells. In addition, we found that CAPON-S influenced the proliferation of glioma cells, possibly through inactivating the Akt signaling pathway. Our findings provide evidence for the roles of CAPON as a potential tumor suppressor gene and a biomarker in glioma.

## 4. Experimental Section

### 4.1. Cell Culture

HEK293T cells and glioma cell lines U87 and C6 were purchased from Shanghai Cell Bank, Type Culture Collection Committee, Chinese Academy of Sciences. The cells were grown in DMEM (293T) or MEM (U87) or 1640 (C6) supplemented with 10% fetal bovine serum (FBS, Hyclone). All cell lines were cultured in a cell incubator containing 5% CO_2_ under the saturated humidity at 37 °C.

### 4.2. Glioma and Nontumorous Human Brain Tissues

We collected human glioma specimens (surgical resection) and nontumorous brain tissues (internal decompression in cerebral trauma) from the Affiliated Hospital of Xuzhou Medical University (Xuzhou, China). Clinico-pathological information of all the subjects was provided in Table S2. Surgically removed tissues were sampled for histological diagnosis and the remaining tissues were immediately frozen and stored in liquid nitrogen for analysis. All glioma specimens had confirmed pathological diagnosis and were classified according to the criteria of World Health Organization (WHO). Informed consents were obtained from all patients and family members and the study was approved by the Research Ethics Committee of Xuzhou Medical University.

### 4.3. Antibodies

Rabbit anti-CAPON antibody was purchased from Santa Cruz Bio. (1:500, Santa Cruz, CA, USA) and could react with both CAPON-L and CAPON-S. Antibodies against GFP (1:100, Sino Biological Inc., Beijing, China), Akt, p-Akt (Thr308 and Ser473), S6, p-S6 (Ser235/236), mTOR and p-mTOR (Ser2448) (1:1000, Cell Signaling Technology, Denver, CO, USA) are commercially available.

### 4.4. RNA Extraction and Quantitative Real Time-PCR (qRT-PCR)

Total RNA was extracted with TRIzol (Invitrogen, Waltham, MA, USA) according to the manufacturer’s protocol and then reversely transcribed into cDNA with the cDNA Reverse transcription kit (Roche, Basel, Switzerland). The target genes were amplified in a final volume of 20 μL with a SYBR Green PCR Maser mix (Roche) and a mixture of forward and reverse primers as follows: human CAPON (Forward: 5′-TGTAGGGTGAGCATGGAATTG-3′; Reverse: 5′-CTAAGTGATTTGGAGCAGCAG-3′), rat CAPON (Forward: 5′-GTGGGCAGCCCCTTAGGTA-3′; Reverse: 5′-GATGCCTGACTCTCGGAACTT-3′) and β-actin (Forward: 5′-CCAACCGCGAGAAGATGA-3′; Reverse: 5′-CCAGAGGCGTACAGGGATAG-3′). PCR temperature cycles were performed as follows: 50 °C for 2 min; 95 °C for 10 min; 45 cycles at 95 °C for 15 s and 60 °C for 1 min. Data were acquired and processed automatically by the Applied Biosystems 7500 Real-Time PCR System. The expression of target genes was normalized by that of the β-actin and the relative absolute amounts of target genes were calculated according to our previous method [26] for statistics.

### 4.5. Western Blot Analysis

Total protein was extracted from the cultured cells or tissues according to the previously described method [27]. Protein concentrations were determined by a BCA Protein Assay Kit (Beyotime, Haimen, China). Fifty microgram protein samples were separated via 10% sodium dodecyl sulfate polyacrylamide gel electrophoresis (SDS-PAGE) and then electrophoretically transferred to a polyvinylidene fluoride (PVDF) membrane (Millipore, Bedford, MA, USA). Membranes were incubated in 3% bovine serum albumin in PBS for 2 h, and then treated with primary antibodies at 4 °C overnight. β-Actin (1:1000, Santa Cruz Bio.) was used as a protein-loading control. In the next day, membranes were incubated in a horseradish peroxidase-labeled goat anti-rabbit/mouse IgG (1:4000, Pierce, Rockford, IL, USA) and detected by the enhanced chemiluminescence detection system (Pierce). Band densities were analyzed using Image J software (National Institute of Health, Bethesda, MD, USA). All fold changes in band densities compared to the normal control group were determined.

### 4.6. Lentivirus Construction, Production, and Infection

In the experiment of overexpression of CAPON-S in U87 cells, the human *capon* variant 2 (Accession number: NM_001126060) was inserted into the pWPXLd plasmid at BamH I and Mlu I sites. The viruses were propagated in 293T cells by cotransfecting the CAPON-S lentivirus plasmid and the helper plasmids. Glioma cell infection was performed with PolyJet (SignaGen, Gaithersburg, MD, USA) according to the manufacturer’s protocol. In 48 h after infection, the virus-infected cells were cultured in the medium containing 2.5 μg/mL puromycin for selection. The surviving cells were used in the subsequent experiments. For silencing CAPON in C6 cells, the shRNA oligos (target sequences: GCCAGCAATATCTTCAGATG for shCAPON-1; GCCTCAGAGTATGAGTCCAA for shCAPON-2) were annealed and then subcloned into the pLL3.7 plasmid at Hpa I and Xho I cloning site. The shCAPON viruses was packaged to infect the C6 cells as described above, so as to establish stably down-regulated cell lines.

### 4.7. Cell Viability Detection

Cell viability was measured with a Cell Counting Kit-8 (CCK-8, Dojindo, Tokyo, Japan). The single cell suspension (5 × 10^3^ /mL, 100 μL) were dispensed in a 96-well plate and cultured for 6, 12, 24, 48, and 72 h, respectively. Then, 10 μL of the CCK-8 reagent was added into each well and incubated for another 1 h. Then the absorbance (value) at 450 nm was measured using a scanning microplate reader. Cell viability at individual time points was normalized to those at 6 h in each group.

### 4.8. EdU Assay

The 5-ethynyl-20-deoxyuridine (EdU) incorporation assay was performed with an EdU assay kit (Ribobio, Guangzhou, China) according to the manufacturer instructions. Cells were seeded into 96-well plates at 1 × 10^4^ cells per well for 24–48 h and then exposed to 50 μM of EdU for 2 h at 37 °C. Subsequently, the cells were fixed with 4% paraformaldehyde, and then permeabilized with 0.5% Triton X-100. Finally, the cells reacted with 100 μL of Apollo^®^ reaction cocktail for 30 min, followed by incubation with 100 μL of Hoechst 33342 (5 μg/mL). The images were taken by an Olympus IX-71 inverted microscope (Tokyo, Japan). The percentage of EdU-positive cells was calculated by dividing the number of EdU-positive cells by the number of Hoechst-stained cells.

### 4.9. Flow Cytometry

The cell cycle was assessed by flow cytometry with a commercial cell cycle analysis kit (NewMed Cytomics, Suzhou, China) according to the manufacturer’s protocol. Briefly, cells were trypsinized into single-cell suspension and collected by centrifugation at 1500 rpm. After washing with PBS for two times, the reagents A, B, and C from the kit were successively added into the cells. The cell suspension was filtered by 50 mm nylon mesh and immediately analyzed by flow cytometry (BD, Franklin Lakes, NJ, USA).

### 4.10. Antibody Array

Analysis of an Intracellular Signaling Array was performed according to the protocol provided by the manufacturer (Cell Signaling Technology). In brief, Vector and CAPON-S glioma cells were lysed in lysis buffer containing 1 mM phenylmethylsulfonyl fluoride. Totally 60 μg of lysate was added onto a slide coated with target-specific capture antibodies. The slide was then incubated with a biotinylated antibody cocktail. Streptavidin-conjugated horseradish peroxidase and LumiGLO reagent were used to visualize the bound detection antibody by chemiluminescence. Phosphorylation or cleavage of important signaling molecules was quantified with the Image J 1.43u (National Institute of Health, Bethesda, MD, USA).

### 4.11. Statistical Analysis

The differences between nontumor group and glioma subgroups were evaluated by one-way ANOVA followed by Dunnett’s multiple comparison. The in vitro data were analyzed by unpaired Student’s *t* test. Quantitative data were obtained from at least three independent experiments and expressed as mean ± S.E.M. Statistical analyses were performed using SPSS version 13.0 (SPSS Inc., Chicago, IL, USA). Tests were two-tailed and values of *p* < 0.05 were considered to be significant.

## Supplementary Materials

Supplementary materials can be found at www.mdpi.com/1422-0067/17/11/1859/s1.
