# Supplementary material for: Low Expression of CAPON in Glioma Contributes to Cell Proliferation via the Akt Signaling Pathway
Source: Int J Mol Sci. 2016 Nov 18;17(11):1859. doi: 10.3390/ijms17111859 (PMC5133859; doi:10.3390/ijms17111859)
Supplement: Supplementary file 1 [file ijms-17-01859-s001.pdf]

Supplementary Materials: Low Expression of CAPON in Glioma Contributes to Cell Proliferation via the Akt Signaling Pathway

Shangfeng Gao, Jie Wang, Tong Zhang, Guangping Liu, Lei Jin, Daofei Ji, Peng Wang, Qingming Meng, Yufu Zhu and Rutong Yu

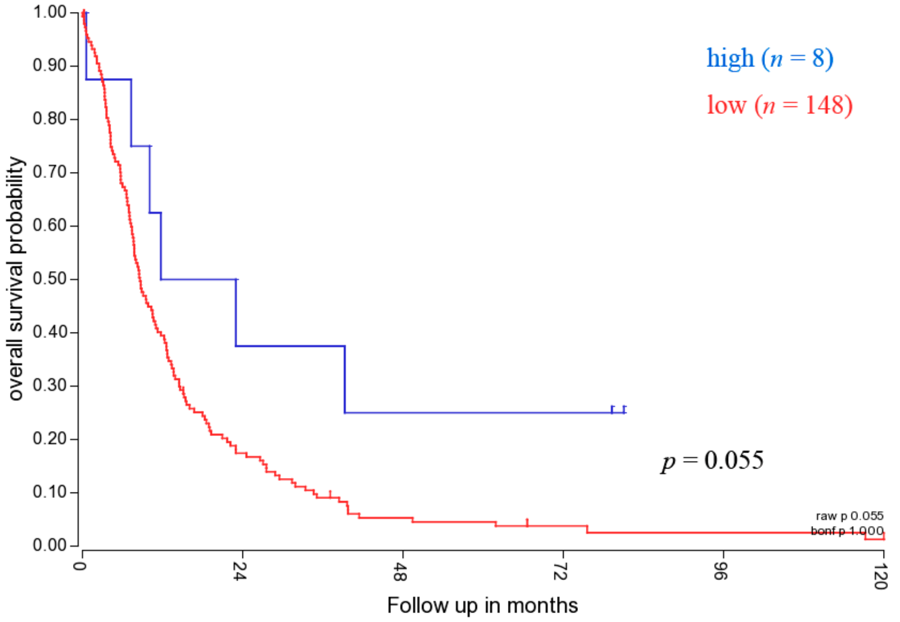

Figure S1. The relationship between the levels of CAPON mRNA and the overall survival in glioblastoma patients. Survival analysis was performed using the Kaplan-Meier survival test in 156 glioblastoma patients based on R2 genomics database (available online: http://hgserver1.amc.nl/cgi-bin/r2/main.cgi).

Table S1. Summary for the antibody array.

No.	Target	Site	Spot Intensity in the CAPON-S Group (Mean ± SEM)	Spot Intensity in the Vector Group (Mean ± SEM)	*p* Value (*t* Test)	*p* Value Summary (*t* Test)
3	Phospho-ERK1/2	Thr202/Tyr204	73.96 ± 6.835 *n* = 4	81.29 ± 2.550 *n* = 4	0.3534	ns
4	Phospho-Stat1	Tyr701	60.76 ± 5.426 *n* = 4	74.67 ± 4.556 *n* = 4	0.0972	ns
5	Phospho-Stat3	Tyr705	62.52 ± 5.969 *n* = 4	78.51 ± 4.925 *n* = 4	0.0843	ns
6	Phospho-Akt	Thr308	60.35 ± 4.169 *n* = 4	73.16 ± 2.602 *n* = 4	0.0403	*
7	Phospho-Akt	Ser473	88.32 ± 7.120 *n* = 4	122.5 ± 7.143 *n* = 4	0.0147	*
8	Phospho-AMPKα	Thr172	85.98 ± 5.095 *n* = 4	83.00 ± 1.784 *n* = 4	0.6006	ns
9	Phospho-S6 Ribosomal Protein	Ser235/236	54.40 ± 3.723 *n* = 4	64.05 ± 2.315 *n* = 4	0.0698	ns
10	Phospho-mTOR	Ser2448	58.44 ± 3.374 *n* = 4	71.72 ± 2.873 *n* = 4	0.0241	*
11	Phospho-HSP27	Ser78	70.85 ± 3.634 *n* = 4	70.62 ± 2.014 *n* = 4	0.9585	ns
12	Phospho-Bad	Ser112	73.41 ± 7.191 *n* = 4	76.35 ± 1.001 *n* = 4	0.6990	ns
13	Phospho-p70 S6 Kinase	Thr389	59.40 ± 4.206 *n* = 4	70.89 ± 3.761 *n* = 4	0.0879	ns
14	Phospho-PRAS40	Thr246	174.7 ± 16.94 *n* = 4	153.9 ± 5.678 *n* = 4	0.2902	ns
15	Phospho-p53	Ser15	54.59 ± 4.561 *n* = 4	57.32 ± 1.281 *n* = 4	0.5859	ns
16	Phospho-p38	Thr180/Tyr182	70.02 ± 3.558 *n* = 4	68.89 ± 2.569 *n* = 4	0.8058	ns
17	Phospho-SAPK/JNK	Thr183/Tyr185	91.56 ± 10.33 *n* = 4	84.66 ± 6.790 *n* = 4	0.5970	ns
18	Cleaved PARP	Asp214	61.07 ± 4.896 *n* = 4	58.54 ± 1.582 *n* = 4	0.6394	ns
19	Cleaved Caspase-3	Asp175	67.25 ± 2.433 *n* = 4	67.15 ± 3.245 *n* = 4	0.9811	ns
20	Phospho-GSK-3β	Ser9	75.34 ± 8.296 *n* = 4	63.55 ± 4.062 *n* = 4	0.2488	ns

ns, no significant differences; * $p < 0.05$, CAPON-S group vs. Vector group.

Table S2. Clinico-pathological information for the studied subjects.

Case No.	Code No.	Gender	Age (Years)	Location	Used for	WHO Grade
1	802681	M	48	Temporal lobe left	WB, qRT-PCR	Nontumor
2	232993	F	59	Frontal lobe left	WB, qRT-PCR	Nontumor
3	774276	F	42	Temporal lobe left	WB, qRT-PCR	Nontumor
4	668280	M	27	Vermis cerebelli	WB, qRT-PCR	Nontumor
5	901447	F	51	Cristae sphenoidalis left	WB, qRT-PCR	Nontumor
6	904118	F	35	Frontal lobe left	WB, qRT-PCR	Nontumor
7	919616	M	57	Cerebelli right	WB, qRT-PCR	Nontumor
8	912226	F	54	Temporal lobe right	WB, qRT-PCR	Nontumor
9	972078	F	49	Not available	WB	Nontumor
10	780411	M	47	Frontal lobe right	WB, qRT-PCR	Grade II
11	797313	F	48	Temporal lobe left	WB, qRT-PCR	Grade II
12	844875	F	63	Not available	WB, qRT-PCR	Grade II
13	859382	F	41	Temporal lobe left	WB, qRT-PCR	Grade II
14	942048	M	21	Occipital lobe left	WB, qRT-PCR	Grade II
15	903335	M	42	Temporal lobe left	WB, qRT-PCR	Grade II
16	857964	F	39	Frontal-parieto lobe left	WB, qRT-PCR	Grade II
17	896722	F	27	Frontal lobe left	WB	Grade II
18	838012	M	42	Temporal lobe right	WB	Grade II
19	811329	F	36	Frontal lobe bilateral	WB, qRT-PCR	Grade II
20	1026751	M	66	Temporal-parieto lobe right	WB	Grade II
21	1019453	F	43	Frontal lobe right	WB	Grade II
22	986708	M	47	Temporal-ocipital lobe left	WB, qRT-PCR	Grade III
23	827030	M	61	Temporal lobe right	WB, qRT-PCR	Grade III
24	912226	F	54	Temporal lobe right	WB, qRT-PCR	Grade III
25	861461	M	57	Temporal-ocipital lobe left	WB, qRT-PCR	Grade III
26	920498	M	50	Temporal-parieto lobe right	WB, qRT-PCR	Grade III
27	761952	F	47	Frontal lobe right	WB, qRT-PCR	Grade III
28	783144	F	57	Frontal lobe right	WB, qRT-PCR	Grade III
29	574549	F	54	Frontal lobe right	WB, qRT-PCR	Grade III
30	835070	F	35	Temporal lobe left	WB	Grade III
31	1029786	M	66	Not available	WB	Grade III
32	1016838	F	45	Cella lateralis	WB	Grade III
33	999737	M	52	Frontal-temporal lobe left	WB	Grade III
34	900637	M	48	Temporal lobe right	WB, qRT-PCR	Grade IV
35	931779	M	53	Frontal lobe left	WB, qRT-PCR	Grade IV
36	953228	F	60	Frontal lobe left	WB, qRT-PCR	Grade IV
37	947804	F	66	Frontal-temporal-parieto lobe left	WB, qRT-PCR	Grade IV
38	813895	F	62	Temporal lobe left	WB, qRT-PCR	Grade IV
39	1033132	F	51	Frontal-temporal-parieto lobe right	WB	Grade IV
40	971853	F	58	Temporal lobe left	WB	Grade IV
41	873610	M	66	Frontal lobe right	WB	Grade IV
42	904534	F	61	Frontal lobe right	WB	Grade IV
43	966838	M	50	Temporal lobe right	qRT-PCR	Grade IV
44	878973	M	51	Temporal lobe left	qRT-PCR	Grade IV
45	879787	F	43	Temporal lobe left	qRT-PCR	Grade IV

F, Female; M, Male; WB, Western blot; qRT-PCR, quantitative real-time PCR; WHO, World Health Organization.
